# The intracellular Ca^2+^ concentration is elevated in cardiomyocytes differentiated from hiPSCs derived from a Duchenne muscular dystrophy patient

**DOI:** 10.1371/journal.pone.0213768

**Published:** 2019-03-15

**Authors:** Fumitoshi Tsurumi, Shiro Baba, Daisuke Yoshinaga, Katsutsugu Umeda, Takuya Hirata, Junko Takita, Toshio Heike

**Affiliations:** Department of Pediatrics, Graduate School of Medicine Kyoto University, Kyoto City, Japan; University of Minnesota Medical Center, UNITED STATES

## Abstract

Duchenne muscular dystrophy (DMD) is the most common and severe form of muscular dystrophy. The major symptoms of this condition are walking difficulties, dyspnea caused by progressive skeletal muscle weakness, and cardiomyopathy. Recent advances in ventilator support devices have dramatically decreased mortality caused by respiratory distress. Consequently, cardiomyopathy resulting in heart failure is currently the major cause of death among DMD patients. One mechanism by which skeletal muscle is damaged in DMD patients involves elevation of the intracellular Ca^2+^ concentration. By contrast, the mechanisms underlying the development of cardiomyopathy are unclear. To investigate this, we examined the intracellular Ca^2+^ concentration and calcium transients in cardiomyocytes differentiated from human induced pluripotent stem cells (hiPSCs). hiPSCs were derived from a DMD patient (DMD-hiPSCs), in whom exon 44 of the gene encoding dystrophin was deleted, and from his parents (control-hiPSCs), who did not carry this mutation. The intracellular Ca^2+^ concentration was measured using the fluorescent indicator indo-1. The fluorescence ratio (410/490 nm) of indo-1 at rest (R_0_), the peak of this ratio (R_max_), and the amplitude (R_max_—R_0_) were significantly higher in cardiomyocytes differentiated from DMD-hiPSCs than in those differentiated from control-hiPSCs. Moreover, mechanical stretching significantly increased the intracellular Ca^2+^ concentration in cardiomyocytes differentiated from DMD-hiPSCs, but not in those differentiated from control-hiPSCs. These findings indicate that elevation of the intracellular Ca^2+^ concentration can cause cardiac damage leading to cardiomyopathy in DMD patients.

## Introduction

Duchenne muscular dystrophy (DMD) is the most common and severe form of muscular dystrophy. This condition is caused by mutations of the gene encoding dystrophin located on chromosome Xp21 and is inherited in an autosomal recessive manner. DMD is relatively common, with an incidence of approximately 1 per 3500 male births. Muscle atrophy and weakness develop progressively with repeated cycles of muscle degeneration and regeneration. The majority of patients begin to experience walking difficulties before puberty. Patients usually develop respiratory muscle failure and heart failure, which are both common lethal complications of DMD, in their late teens and early twenties [1].

Until about 1990, respiratory insufficiency and respiratory tract infections were the major causes of death among DMD patients [2–4]. However, this changed after the development of ventilator support devices, such as nocturnal home ventilation [5,6]. The mean lifespan of non-ventilated DMD patients was 14.4 years in the 1960s, whereas that of ventilated DMD patients was 25.3 years in the 1990s [6], and it is currently 36.23 years. Cardiomyopathy is now the major cause of death among DMD patients. The percentage of DMD patients dying from cardiac problems increased from 8% to 44% after the 1990s [7].

Cardiomyopathy associated with fibrosis usually leads to dilated cardiomyopathy (DCM). In total, 25% and 59% of DMD patients aged less than 6 years and 6–10 years have a sub-clinical stage of DCM, respectively [8]. More than 80% of DMD patients older than 18 years have reduced cardiac functions [9,10]. Moreover, 90% of DMD patients develop DCM [1].

Patients with DMD lack dystrophin, a major structural protein in muscle cells. Dystrophin links the cytoskeleton with the extracellular matrix in muscles [11]. Muscle contraction leads to rupture of the sarcolemma in *mdx* mice, an animal model of DMD [12]. In addition, Franco et al. reported that muscles of *mdx* mice contain mechano-transducing ion channels that open in response to stretching, leading to abnormal leakage of Ca^2+^ into cells [13]. Elevation of the intracellular Ca^2+^ concentration is thought to be important for the initiation of skeletal muscle damage in DMD patients based on findings obtained using *mdx* mice [14]. An elevated Ca^2+^ concentration leads to cell damage in these mice [15,16]. Abnormal handling of intracellular Ca^2+^ is also observed in cardiomyocytes of patients with end-stage heart failure [17].

Here, we investigated the development of cardiomyopathy in DMD patients by examining calcium transients in cardiomyocytes differentiated from human induced pluripotent stem cells (hiPSCs).

## Materials and methods

### Generation of hiPSCs from fibroblasts

Human iPS cells experiments were approved by the Ethics Committee of Kyoto University (approval number: R0091, G259), and it was conducted according to the principles expressed in the Declaration of Helsinki. A written informed consent was obtained from the parents. hiPSCs were established by transducing fibroblasts obtained from a DMD patient and his parents with Yamanaka factors (*Oct3/4*, *Sox2*, *Klf4*, and *c-Myc*). These hiPSCs were maintained on dishes coated with Matrigel (14.6 μg/cm^2^; Becton, Dickinson and Company) in mTeSR1 (Stem Cell Technologies) and passaged every 4–5 days. Cells were treated with 5 mM Y-27632 (Nacalai Tesque), a ROCK inhibitor, for 1 day after passage to prevent apoptosis.

### Differentiation and purification of cardiomyocytes

Differentiation of hiPSCs into cardiomyocytes was induced by the matrix sandwich method as previously described [18,19] with some modifications. Briefly, hiPSCs derived from the DMD patient (DMD-hiPSCs) and his parents (control-hiPSCs) were passaged onto 12-well plates coated with growth factor-reduced Matrigel (8.7 μg/cm^2^; Becton, Dickinson and Company) in mTeSR1 (Stem Cell Technologies). Cells were overlaid with growth factor-reduced Matrigel when they reached 80–90% confluency. On day 5 of differentiation, the culture medium was replaced by RPMI medium (Invitrogen) containing B27 supplement (Invitrogen) and 100 ng/mL Dkk-1 (R&D Systems) for 2 days. After approximately 30–40 days, cardiomyocytes were purified by magnetic-activated cell sorting (Miltenyi Biotec K.K.) using an antibody against vascular cell adhesion molecule-1 (BioLegend).

### PCR analysis

Genomic DNA was isolated from fibroblasts obtained from the DMD patient and his parents. Exons 43, 44, and 45 of the gene encoding dystrophin were analyzed by PCR. The cycling conditions were as follows: 40 cycles of denaturation at 95°C for 5 min, annealing at 55°C for 30 sec, and extension at 72°C for 1 min, and a final extension at 72°C for 7 min. The following primers were used: 43 forward, tgc aac acc att tgc tac c; 43 reverse, atc att tct gca agt atc aag; 44 forward, gtt act tga aac taa act ctg caa atg; 44 reverse, aca aca aca gtc aaa agt aat ttc cat c; 45 forward, ttc ttt gcc agt aca act gc; and 45 reverse, tct gct aaa atg ttt tca ttc c.

### Immunostaining

Cells were fixed in phosphate buffered saline (PBS) containing 4% paraformaldehyde for 15 min at room temperature, washed with PBS, and treated with PBS containing 0.1% Triton X-100 and 5% goat or donkey serum for 30 min at room temperature. Thereafter, cells were stained with the following primary antibodies for 1 h at room temperature or overnight at 4°C: anti-NANOG (1:100, R&D Systems), anti-TRA-1-81 (1:100, Millipore), anti-cardiac troponin T (cTnT; 1:200, Thermo Scientific), and anti-dystrophin (1:100, MANDRA1, Sigma-Aldrich). Next, cells were stained with the following secondary antibodies for 1 h at room temperature: Cy3-conjugated donkey anti-mouse IgM (1:500, Jackson ImmunoResearch), Alexa Fluor 488-conjugated donkey anti-goat IgG (1:500, Invitrogen), Alexa Fluor 568-conjugated donkey anti-mouse IgG (1:500, Invitrogen) and a Zenon Alexa Fluor 488 labelling kit (Invitrogen). Nuclei were counterstained with 10 μg/mL Hoechst 33342 (Invitrogen). Images were acquired using a fluorescence microscope (BZ-X700 or BZ-X710, Keyence). Confocal images were captured by TCS SP8 confocal microscope (Leica Microsystems).

### Karyotypes

The karyotype of each hiPSC line was analyzed by Nihon Gene Research Laboratories Inc.

### RT-PCR

Total RNA was isolated from hiPSCs using an RNeasy Mini Kit (Qiagen) and treated with DNase. Thereafter, 1 μg of total RNA was reverse-transcribed into cDNA using an Omniscript RT Kit (Qiagen). Quantitative PCR analysis of *Oct3/4*, *Sox2*, *Klf4*, and *c-Myc* was performed on an ABI Prism 7900HT Sequence Detection System (Applied Biosystems) using SYBR Premix Ex Taq II (Takara Bio). The established human embryonic stem cell line KhES-1 [20] was analyzed as a control.

### Teratoma formation

Animal experiments were approved by the Institutional Review Board (IRB) of Kyoto University (approval number: Med Kyo 12556). Undifferentiated hiPSCs were treated with collagenase IV (Invitrogen), collected in tubes, and centrifuged. The pellets were resuspended in mTeSR1 (Stem Cell Technologies) containing 5 mM Y-27632 (Nacalai Tesque). Under general anesthesia with Sevoflurane, approximately 3 million cells were injected into the testis of a NOD/scid/γc null mouse (Central Institute for Experimental Animals). Eight weeks later, tumors were harvested after euthanasia by carbon dioxide, dissected into 1 cm^3^ cubes, and fixed in PBS containing 4% paraformaldehyde. Paraffin-embedded tissue was sliced and stained with hematoxylin and eosin.

### Measurement of the intracellular Ca^2+^ concentration

The intracellular Ca^2+^ concentration was measured using the fluorescent indicator indo-1 (Dojindo Molecular Technologies) and a video image analysis system (AQUACOSMOS, Hamamatsu Photonics). Cardiomyocytes differentiated from hiPSCs were re-plated onto 12 mm glass bottom dishes (Iwaki) and incubated for 2–3 days to allow cell adhesion. Thereafter, cells were loaded with 3 μM indo-1 for 30 min. Prior to recording, the dish was washed with PBS and filled with Tyrode’s salt solution (Sigma-Aldrich). The fluorescence ratio (410/490 nm) of indo-1 was plotted along the y axis. This ratio at rest (R_0_), the peak of this ratio (R_max_), and the amplitude (R_max_—R_0_) were measured.

### Mechanical stretching of cultured cardiomyocytes

To expose cardiomyocytes to mechanical stress, cardiomyocytes differentiated from hiPSCs were re-plated onto fibronectin-coated silicone chambers (Strex Inc.) and incubated for 2–3 days to allow cell adhesion. Thereafter, mechanical stress was induced by mechanically stretching cardiomyocytes (120% elongation) at a frequency of 60 cycles per min using an automatic stretching system STB-140 (Strex Inc.). The intracellular Ca^2+^ concentration was recorded prior to and 3 h after stretching by measuring indo-1 fluorescence.

### Statistical analysis

Results are presented as mean ± standard error of the mean. Statistical analyses were performed using a one-way ANOVA followed by the Tukey-Kramer test with JMP^®^ Pro (11.0.0) software. *p* values less than 0.05 were considered statistically significant.

## Results

### Clinical history of the DMD patient and generation of hiPSCs

**Fig 1:**
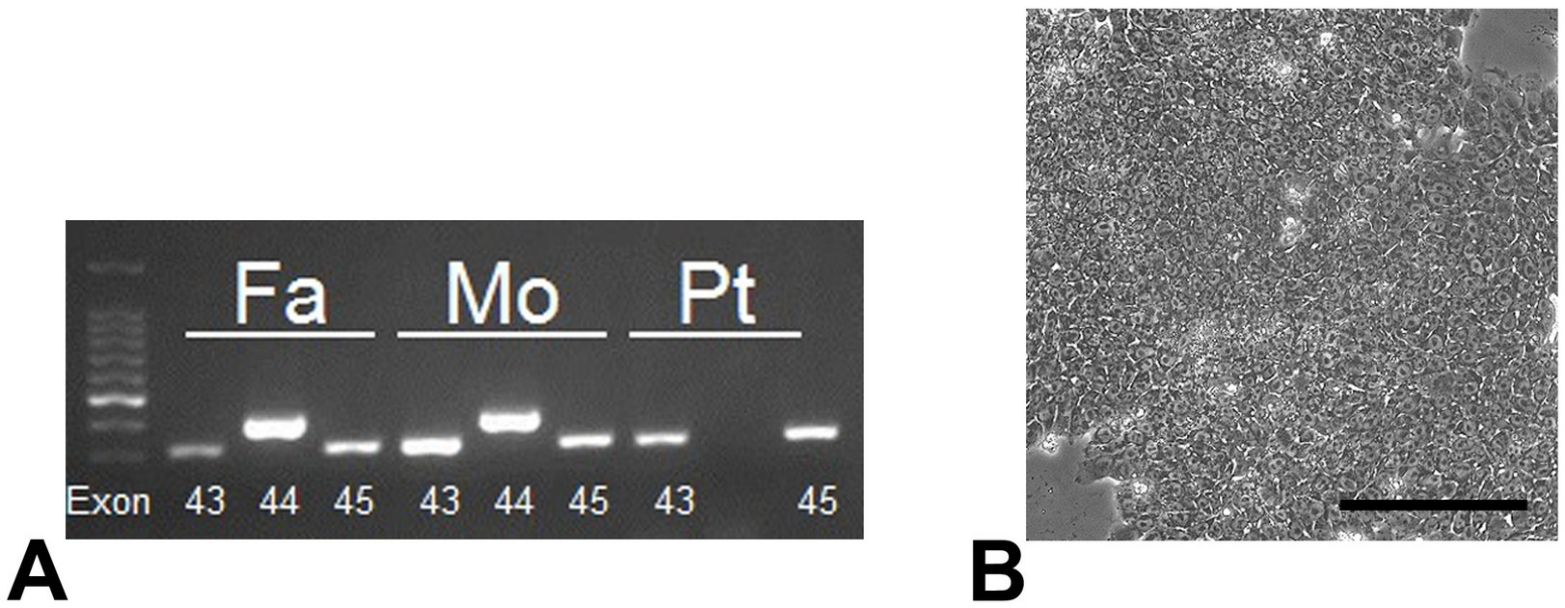
Mutational analysis and generation of hiPSCs. (A) PCR analysis of exons 43, 44, and 45 of the gene encoding dystrophin in fibroblasts. Fa, father; Mo, mother; and Pt, patient. (B) Morphology of a colony of DMD-hiPSCs. Scale bar: 100 μm.

A 3-year-old boy diagnosed with DMD presented to our hospital. He had undergone an adenoidectomy at 2 years of age. A preoperative blood test demonstrated that his levels of serum muscle enzymes (i.e., aspartate aminotransferase, alanine aminotransferase, and creatine phosphokinase) were significantly elevated, indicative of a muscular disease. Genetic analysis revealed that exon 44 of the gene encoding dystrophin was deleted in the patient. There was no family history of muscular diseases, and the patient’s parents did not carry mutations of this gene. Deletion of exon 44 was confirmed by PCR analysis of cultured fibroblasts isolated from the patient (Fig 1A). This is a high-risk deletion that leads to lethal DCM[21]. The cardiac function of the patient gradually deteriorated, and he started taking anti-heart failure drugs when 11 years old.

hiPSCs were generated by retrovirally transducing fibroblasts isolated from the skin of the patient and his parents with *Oct3/4*, *Sox2*, *Klf4*, and *c-Myc*. These hiPSCs formed colonies, and all cells were cuboid and tightly packed in each colony (Fig 1B). We established two hiPSC lines derived from the patient (DMD-1 and DMD-2), one derived from his father (Con-1), and one derived from his mother (Con-2).

### hiPSCs are pluripotent *in vitro* and *in vivo*

**Fig 2:**
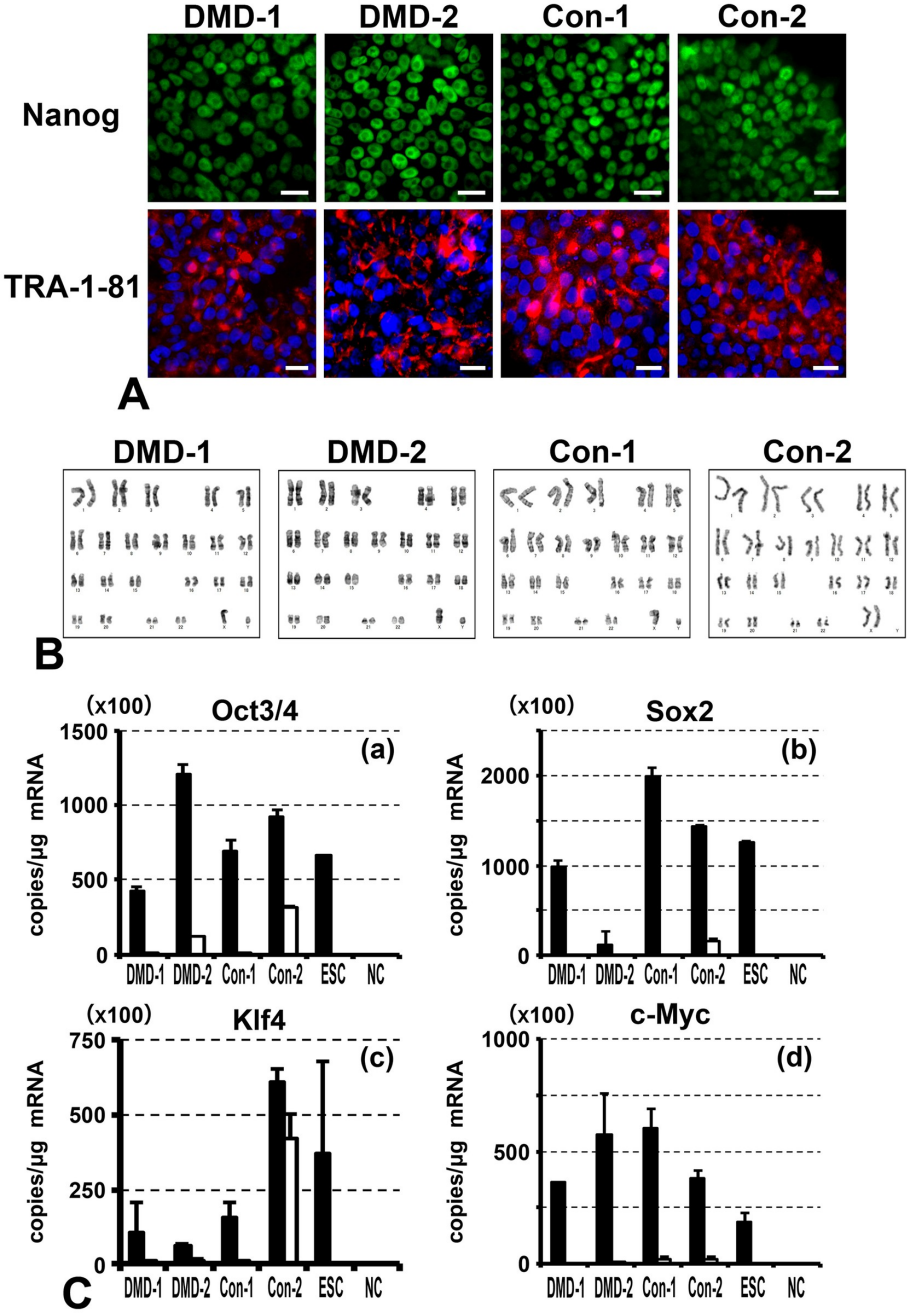
Characterization of hiPSCs. (A) Immunostaining of the pluripotency markers NANOG (green) and TRA-1-81 (red) in hiPSCs. Nuclei were counterstained with Hoechst 33342 (blue). Scale bar: 20 μm. (B) Karyotyping of hiPSCs. (C) Quantitative PCR analysis of total and exogenous *Oct3/4*, *Sox2*, *Klf4*, and *c-Myc* expression in hiPSCs. Black and white bars indicate total and exogenous gene expression, respectively. ESC: human embryonic stem cell, NC: negative control (water).

**Fig 3:**
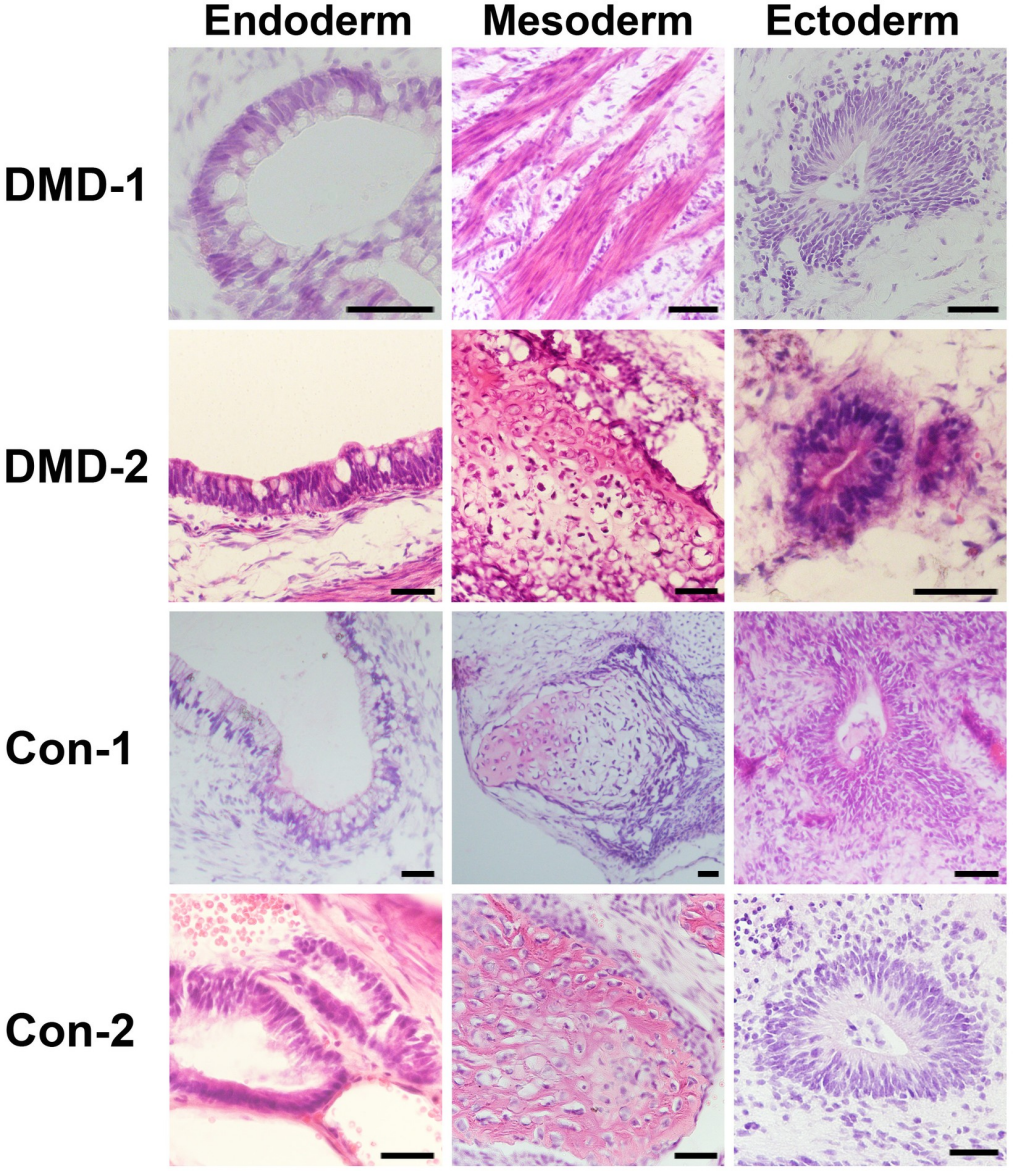
Formation of teratomas by hiPSCs. Hematoxylin and eosin staining demonstrated that teratomas formed by each hiPSC line contained all three germ layers. Gut-like tissues are observed in each endoderm panel. Muscular tissues are observed in the mesoderm panels of DMD-1 hiPSCs, while cartilage tissues are observed in those of DMD-2, Con-1, and Con-2 hiPSCs. Neural rosette formation is observed in each ectoderm panel. Scale bar: 50 μm.

The generated hiPSCs were characterized by immunostaining for pluripotency markers, karyotyping, assessing exogenous gene silencing, and examining teratoma formation. All the hiPSC lines expressed NANOG and TRA-1-81 (Fig 2A) and had a normal karyotype (Fig 2B). Quantitative PCR analysis demonstrated that all the exogenous genes were silenced in each hiPSC line, with the exception of *Oct3/4* and *Klf4* in Con-2 hiPSCs (Fig 2C). Hematoxylin and eosin staining showed that each hiPSC line differentiated into all three germ layers (endoderm, mesoderm, ectoderm) *in vivo* (Fig 3). Although exogenous *Oct3/4* and *Klf4* were still expressed in Con-2 hiPSCs, expression of endogenous *Oct3/4* and *Klf4* was increased in these cells and they formed teratomas. Thus, all the hiPSC lines were undifferentiated and pluripotent.

### Cardiomyocytes differentiated from hiPSCs express mature cardiac markers

**Fig 4:**
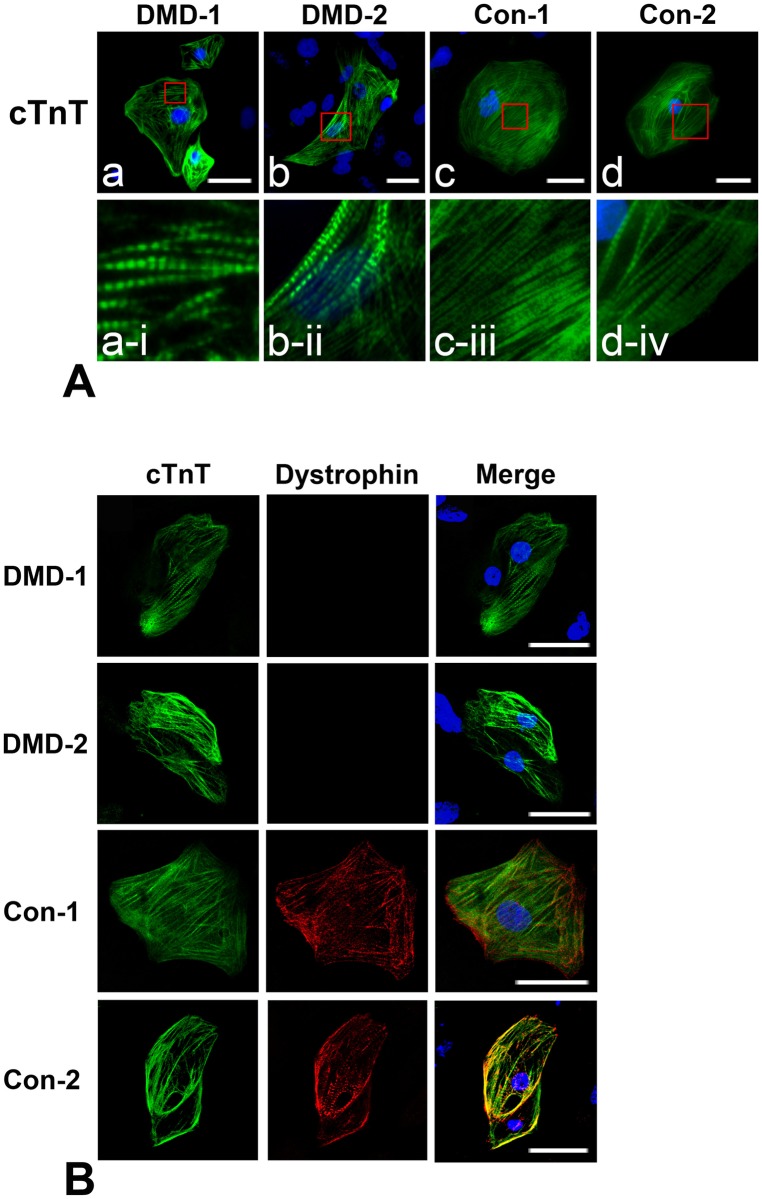
Immunostaining of cardiomyocytes differentiated from hiPSCs. (A) (a—d) Cardiomyocytes differentiated from hiPSCs expressed cTnT (green). Nuclei were counterstained with Hoechst 33342 (blue). Scale bar: 20 μm. (a-i—d-iv) Magnified images in red squares in panels of (a—d). (B) Immunostaining of dystrophin (red) and cTnT (green). Dystrophin was not detected in cardiomyocytes differentiated from DMD-1 and -2 hiPSCs. Nuclei were counterstained with Hoechst 33342 (blue). Scale bar: 20 μm.

The hiPSC lines were differentiated into cardiomyocytes, and these cells were purified by magnetic-activated cell sorting using an antibody against vascular cell adhesion molecule-1. The cardiomyocytes expressed cTnT and displayed sarcomeric structures (Fig 4A). Staining with an anti-dystrophin antibody demonstrated that dystrophin was present mainly on cytoplasmic membranes of cardiomyocytes differentiated from Con-1 and -2 hiPSCs, but not in the cytosol of cardiomyocytes differentiated from DMD-1 and -2 hiPSCs (Fig 4B). These results indicate that cardiomyocytes differentiated from DMD-1 and -2 hiPSCs structurally recapitulate those found in DMD patients.

### The intracellular Ca^2+^ concentration is elevated in cardiomyocytes differentiated from DMD-hiPSCs

**Fig 5:**
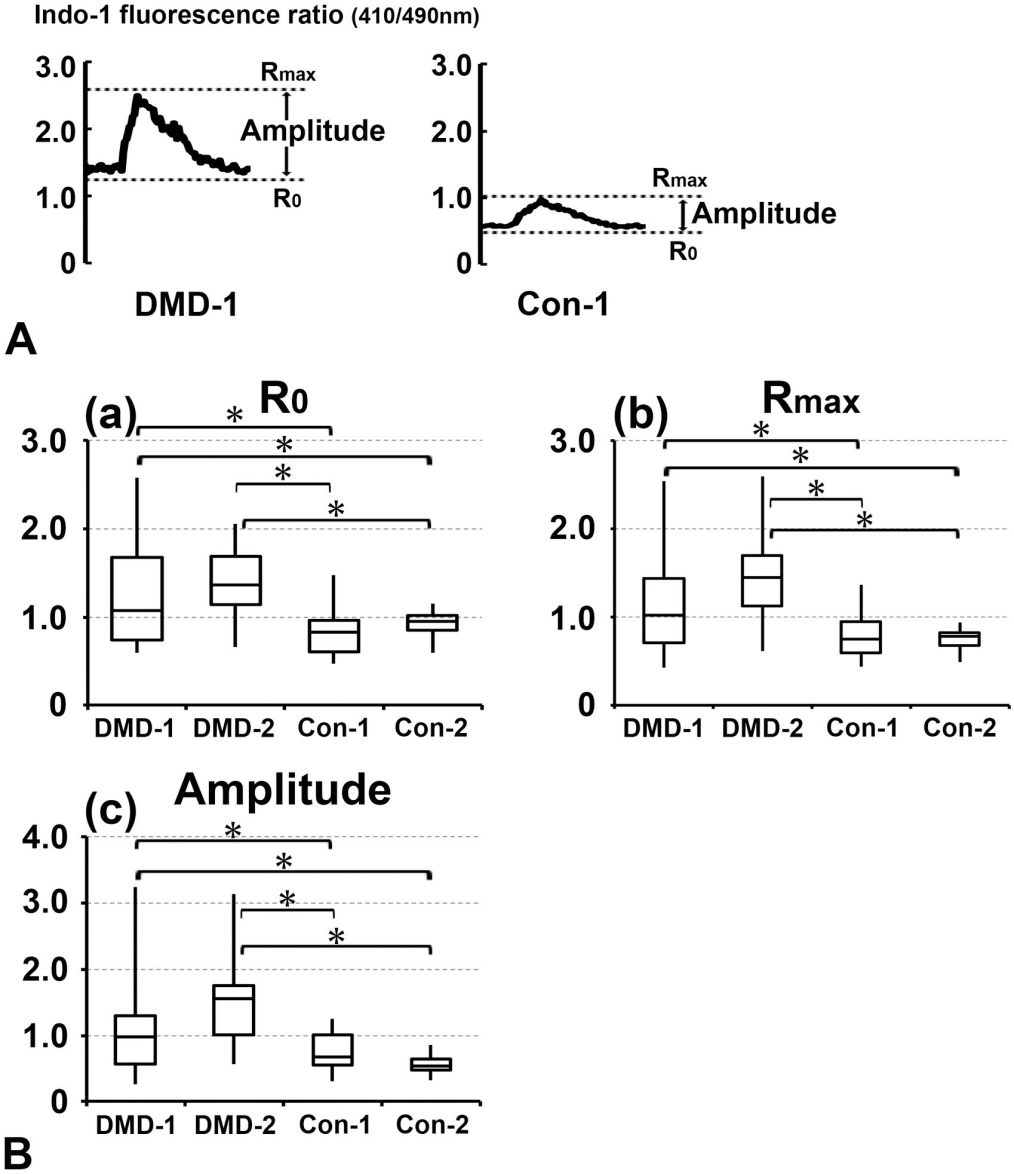
Measurement of the intracellular Ca^2+^ concentration using the fluorescent indicator Indo-1. (A) Representative calcium transients. The fluorescence ratio (410/490 nm) of indo-1 is plotted along the y axis. This ratio at rest (R_0_), the peak of this ratio (R_max_), and the amplitude (R_max_—R_0_) were measured. (B) Intracellular Ca^2+^ concentration. The indo-1 fluorescence ratio (410/490 nm) at rest (R_0_), the peak of this ratio (R_max_), and the amplitude (R_max_—R_0_) were measured in cardiomyocytes. n = 12 (DMD-1), n = 12 (DMD-2), n = 30 (Con-1), and n = 28 (Con-2). *, *p*<0.05.

**Fig 6:**
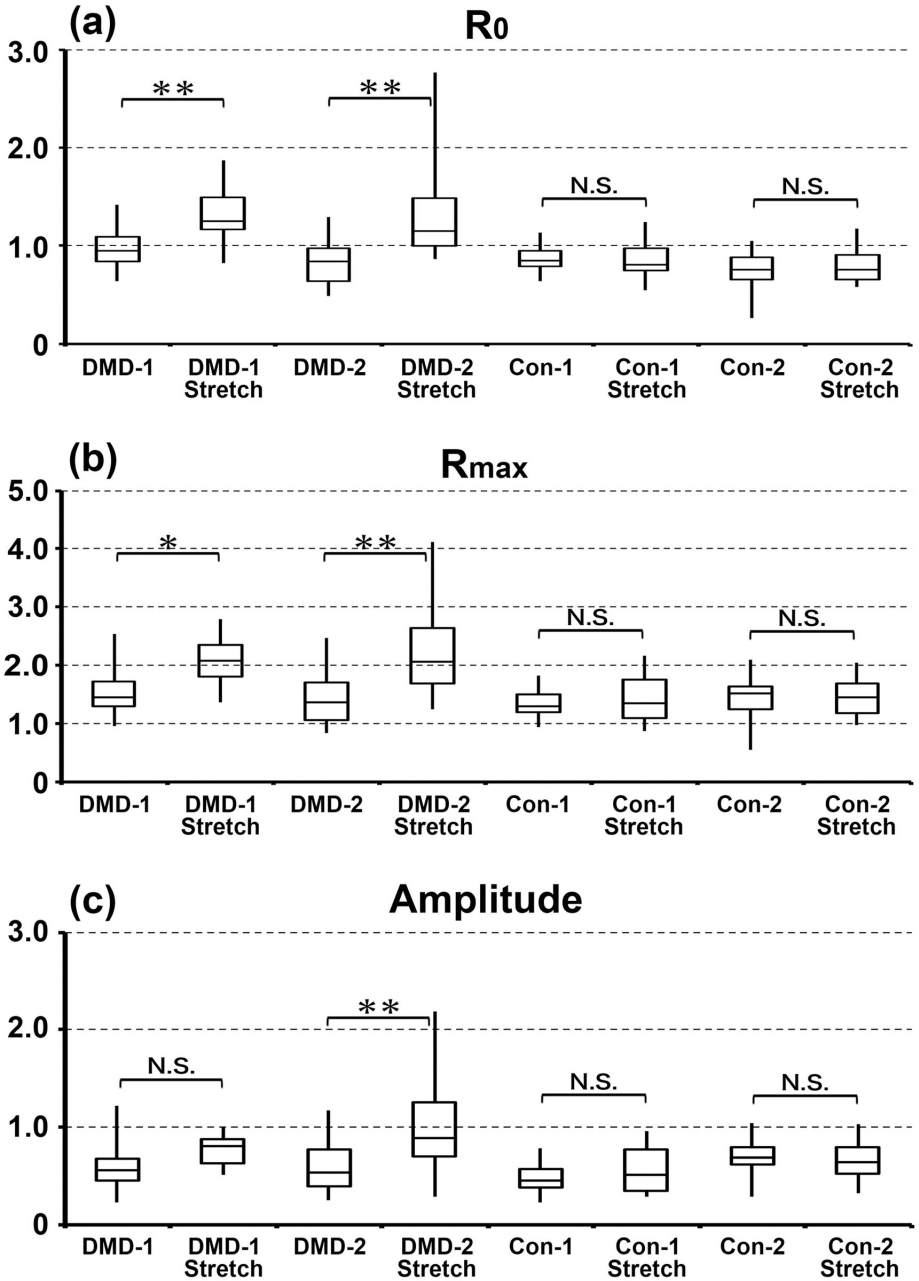
The intracellular Ca^2+^ concentration before and after mechanical stretching. The fluorescence ratio (410/490 nm) of indo-1 at rest (R_0_), the peak of this ratio (R_max_), and the amplitude (R_max_—R_0_) were measured before and after mechanical stretching. n = 26 (DMD-1), n = 16 (DMD-1 Stretch), n = 32 (DMD-2), n = 30 (DMD-2 Stretch), n = 26 (Con-1), and n = 14 (Con-1 Stretch), n = 30 (Con-2), and n = 16 (Con-2 Stretch). **, *p*<0.01; *, *p*<0.05; and N.S., not significant.

The intracellular Ca^2+^ concentration was measured using the fluorescent indicator indo-1. Representative calcium transients of cardiomyocytes derived from DMD- and control-hiPSCs are shown in Fig 5A. During the recording, beat rates of DMD-1, DMD-2, Con-1 and Con-2 cardiomyocytes were 8.5±2.1, 20.9±2.1, 9.6±1.3, 5.7±1.4 per minute, respectively. The fluorescence ratio (490/410 nm) of indo-1 indicates the intracellular Ca^2+^ concentration, and the curve represents the change in this concentration. The indo-1 fluorescence ratio at rest (R_0_), the peak of this ratio (R_max_), and the amplitude (R_max_—R_0_) were significantly higher in cardiomyocytes differentiated from DMD-1 and -2 hiPSCs than in those differentiated from Con-1 and -2 hiPSCs (Fig 5Ba–5Bc). R_0_ of DMD-1, DMD-2, Con-1 and Con-2 cardiomyocytes were 1.26±0.10, 1.40±0.10, 0.83±0.06, 0.93±0.06 (mean ± SE), respectively. R_max_ of DMD-1, DMD-2, Con-1 and Con-2 cardiomyocytes were 2.40±0.21, 2.88±0.21, 1.56±0.13, 1.49±0.14 (mean ± SE), respectively. Amplitude of DMD-1, DMD-2, Con-1 and Con-2 cardiomyocytes were 1.15±0.13, 1.48±0.13, 0.74±0.08, 0.57±0.08 (mean ± SE), respectively. There was no correlation between beat rate and R_0_, Rmax, Amplitude by calculating correlation coefficients (HR and R_0_ (p = 0.0965), HR and Rmax (p = 0.6744), HR and Amplitude (p = 0.5900). Next, cardiomyocytes differentiated from DMD-1, -2 and Con-1, -2 hiPSCs were mechanically stretched to investigate basal intracellular Ca^2+^ concentration and intracellular Ca^2+^ reactions to mechanical stress. Stretching significantly increased the indo-1 fluorescence ratio at rest (R_0_) and the peak of the ratio (R_max_) in cardiomyocytes differentiated from DMD-1 and -2 hiPSCs, but not in cardiomyocytes differentiated from Con-1 and -2 hiPSCs (Fig 6a–6c). However, amplitude (R_max_—R_0_) was significant difference only in cardiomyocytes differentiated from DMD-2 hiPSCs. R_0_ of DMD-1, DMD-1 stretch, DMD-2, DMD-2 stretch, Con-1, Con-1 stretch, Con-2 and Con-2 stretch cardiomyocytes were 0.97±0.05, 1.29±0.06, 0.82±0.05, 1.33±0.05, 0.87±0.05, 0.87±0.07, 0.75±0.05, 0.80±0.06 (mean ± SE), respectively. R_max_ of DMD-1, DMD-1 stretch, DMD-2, DMD-2 stretch, Con-1, Con-1 stretch, Con-2 and Con-2 stretch cardiomyocytes were 1.56±0.09, 2.06±0.11, 1.40±0.08, 2.29±0.08, 1.34±0.09, 1.43±0.12, 1.44±0.08, 1.46±0.11 (mean ± SE), respectively. Amplitude of DMD-1, DMD-1 stretch, DMD-2, DMD-2 stretch, Con-1, Con-1 stretch, Con-2 and Con-2 stretch cardiomyocytes were 0.59±0.06, 0.77±0.07, 0.58±0.05, 1.03±0.05, 0.47±0.06, 0.56±0.08, 0.69±0.05, 0.66±0.07 (mean ± SE), respectively. These results indicate that the intracellular Ca^2+^ concentration, especially basal concentration, is much higher in cardiomyocytes differentiated from DMD-hiPSCs than in those differentiated from control-hiPSCs. Moreover, this difference is larger after stretching than before stretching only in cardiomyocytes differentiated from DMD-hiPSCs.

## Discussion

DMD is the most common and severe form of muscular dystrophy. Patients with this condition lack dystrophin, a major structural protein in muscle cells. Muscular atrophy resulting in muscle weakness develops progressively. DMD can also lead to DCM. Recent reports revealed that the percentage of DMD patients who die due to cardiac problems has rapidly increased, while the percentage who die due to respiratory complications has concomitantly decreased.

Dystrophin links the cytoskeleton with the extracellular matrix in muscles [10], which helps to maintain the shape of muscle cells. Consequently, changes in muscle cell morphology were thought to mainly underlie the progressive muscle weakness observed in DMD patients. Repeated stretch-contraction cycles were considered to cause rupture of the sarcolemma [11]. However, recent studies of *mdx* mice revealed another mechanism underlying progressive skeletal muscle weakness in DMD patients. The absence of dystrophin disrupts signal transduction to a type of mechano-transducing ion channel and consequently muscle stretching leads to abnormal leakage of Ca^2+^ into skeletal muscle cells [12]. Elevation of the intracellular Ca^2+^ is thought to be important for initiation of the pathogenesis of DMD [13] and damages cells [14,15]. Abnormal handling of Ca^2+^ is observed in patients with end-stage heart failure [16]. The severity of cardiomyopathy does not correlate with the severity of skeletal muscle damage in all DMD patients[22]. To investigate the mechanisms by which cardiomyopathy develops in DMD patients, we measured the intracellular Ca^2+^ basal concentration and change in cardiomyocytes differentiated from DMD-hiPSCs, which recapitulated the phenotypes of DMD *in vitro*. Exon 44 of the gene encoding dystrophin was deleted in the DMD patient, which causes severe DCM[21].

hiPSCs were first generated by Yamanaka et al. in 2007[23]. These cells are promising tools not only for regenerative medicine, but also for developing *in vitro* disease models, investigating the pathogenesis of diseases, and discovering new drugs[24]. In addition, hiPSCs can be easily differentiated into various cell types *in vitro*.

Measurement of the fluorescence ratio (410/490 nm) of indo-1 demonstrated that the intracellular Ca^2+^ concentration was much higher in cardiomyocytes differentiated from DMD-hiPSCs than in those differentiated from control-hiPSCs. This result is similar to the findings of a previous report concerning skeletal muscle of *mdx* mice [12]. Mechanical stretching increased the intracellular Ca^2+^ concentration in cardiomyocytes differentiated from DMD-hiPSCs, but not in those differentiated from control-hiPSCs. Especially, the elevation of R_0_ value means that reticulums in cardiomyocytes cannot fully re-intake the Ca^2+^ which was emitted during contraction phase. Under this idea, DMD cardiomyocytes have diastolic dysfunction, because their R_0_ value is higher than R_0_ value of control cardiomyocytes both before and after mechanical stretch stress. Given the previous report of a relationship between calcium transients and heart failure [16], calcium transients in cardiomyocytes may trigger cardiomyopathy in DMD patients.

In conclusion, calcium overload is one way by which cardiomyopathy develops in DMD patients. Modulation of calcium transients in cardiomyocytes may be a useful strategy to treat cardiomyopathy in DMD patients and thereby improve their prognosis.

## Conclusion

Calcium overload might be one mechanism by which cardiomyopathy develops in DMD patients. Reducing calcium overload is a potential strategy to treat cardiomyopathy in such patients.
